# Improving the Analysis of E-Cigarette Emissions: Detecting Human “Dry Puff” Conditions in a Laboratory as Validated by a Panel of Experienced Vapers

**DOI:** 10.3390/ijerph182111520

**Published:** 2021-11-02

**Authors:** Wouter F. Visser, Erna J. Z. Krüsemann, Walther N. M. Klerx, Karin Boer, Naomi Weibolt, Reinskje Talhout

**Affiliations:** 1 Centre for Health Protection, National Institute for Public Health and the Environment (RIVM), Antonie van Leeuwenhoeklaan 9, 3721 MA Bilthoven, The Netherlands; ernakrusemann@hotmail.com (E.J.Z.K.); walther.klerx@rivm.nl (W.N.M.K.); karin.boer@rivm.nl (K.B.); naomi.weibolt@rivm.nl (N.W.); reinskje.talhout@rivm.nl (R.T.); 2 Division of Human Nutrition and Health, Wageningen University, P.O. Box 17, 6700 AA Wageningen, The Netherlands

**Keywords:** e-cigarette, dry puff, emissions, chemical analysis

## Abstract

Introduction: E-cigarette product regulation requires accurate analyses of emissions. User behavior, including device power setting selection, should be mimicked closely when generating e-cigarette emissions in a laboratory. Excessively high power settings result in an adverse burnt off-taste, called “dry puff flavor”. This should be avoided because it results in an overestimation of toxicant levels (especially certain carbonyls). This study presents a human volunteer-validated approach to detect excessively high e-cigarette power settings by HPLC-DAD (high-performance liquid chromatography—diode array detection) carbonyl analysis. Methods: Thirteen experienced e-cigarette users evaluated whether the “dry puff flavor” was present at different power settings (10 W–25 W), recording their assessment on a 100-unit visual analog scale (VAS). They assessed e-cigarettes equipped with 1.2 Ω or 1.6 Ω coils containing menthol, vanilla or fruit-flavored e-liquids. In a machine-vaping experiment, emissions from the same liquid/coil/power setting combinations were subjected to HPLC-DAD analysis of dinitrophenol hydrazine (DNPH)-derivatized carbonyls, such as lactaldehyde and formaldehyde. A simple algorithm, based on the cutoff values for each marker, was applied to relate the dry puff flavor (as assessed by the human volunteers) to the laboratory measurements. Results: Eleven carbonyl compounds were found to agree with the human assessments. Based on the amounts of these compounds in the emissions, the dry-puff flavor did match at all combinations of e-liquids and coils examined. Dry-puff flavor was observed at different power levels with the different liquids tested. Conclusions: The described method can detect dry puff conditions and is therefore a useful tool to ensure user-relevant conditions in laboratory analyses of e-cigarette emissions. Implications: This study improves the chemical analysis of e-cigarette emissions. It offers a method to select an appropriate (i.e., user-relevant) power setting for e-cigarettes, which is a critical parameter for emission analysis and therefore important for regulatory purposes and risk assessments. Compared to the approach of using human volunteers to select appropriate power settings for different products by taste, the described method is cheaper, faster, more practical and more ethical.

## 1. Introduction

E-cigarettes have established a considerable market share over the last decade [1,2]. The continued popularity of these products warrants the development of accurate laboratory methods for analyzing e-cigarette emissions. For example, assessments of the health risks associated with e-cigarettes rely heavily on measurements of the levels of harmful components in the emissions. Many studies have been published on this subject, and the majority of researchers agree that e-cigarettes are less harmful than regular tobacco cigarettes. However, some uncertainty still exists, especially regarding the relative magnitude of the health risks of e-cigarette use (compared to that of other tobacco products, such as cigarettes) and the long-term effects of e-cigarette use [3,4,5,6].

Product regulation contributes to reducing the health risks associated with the use of tobacco and related products such as e-cigarettes [7]. Many countries have implemented regulation that limits or prohibits the use of certain ingredients or the presence of impurities in e-liquids that may contribute to the toxicity, attractiveness or addictiveness of e-cigarettes. For instance, tobacco-specific nitrosamine (TSNA) impurities in e-cigarette refill liquids are regulated in the Netherlands, because the European Tobacco Product Directive (TPD) [8] specifies that “only ingredients are used in the nicotine-containing liquid that do not pose a risk to human health in heated or unheated form” and “only ingredients of high purity are used in the manufacture of the nicotine-containing liquid”. Developing and enforcing product regulation requires accurate laboratory methods for the analysis of e-liquids and e-cigarette emissions.

A critical step in analyzing e-cigarette emissions in the laboratory is the generation and collection of emission samples using an automated vaping machine. Vaping machines take puffs according to a preprogrammed puffing topography (i.e., puff duration, volume, profile and interval). The composition of e-cigarette emissions is a function of this topography [9], and it is therefore important to select a user-relevant topography. Many studies have been conducted to study the topography of e-cigarette users [10,11,12,13,14], which is complex and variable between users and between products with different characteristics, such as nicotine content and flavor [14]. Despite these challenges, standardized vaping topographies have been proposed that are intended to be relevant for the analysis of e-cigarette emissions in the laboratory [15,16].

Another critical parameter when collecting e-cigarette emissions for laboratory analysis is the e-cigarette power or temperature [17,18,19,20,21,22,23], Many current e-cigarette models feature user-adjustable power settings [24]. At excessively high power settings, the high temperature of the e-cigarette heating element leads to increased thermal decomposition of the e-liquid ingredients [21], resulting in the formation of excessively high levels of harmful compounds, particularly carbonyls [18,19,20,21,22,23,25,26]. Farsalinos et al. [26] reported that formaldehyde, acetaldehyde and acrolein levels were raised by 30–250 times when using excessively high power settings. A highly adverse burnt flavor occurs when the power setting is too high (or when the heating element runs dry for other reasons), which is known among vapers as a “dry puff” or “dry hit” [25]. Users avoid the bad flavor by lowering the power setting of their device and by maintaining a sufficient level of e-liquid. It is therefore important to avoid dry puffs in the laboratory, mimicking the behavior of users. Failure to account for this effect may lead to considerable overestimations of the toxicity of e-cigarette emissions in the assessment of e-cigarette health risks. For instance, Jensen et al. [27] estimated that the lifetime cancer risk of e-cigarette use due to formaldehyde exposure is 15-times higher than that of smoking tobacco cigarettes, but this study has been met with strong criticism for using excessively high power settings [28].

E-cigarette users recognize dry puffs by taste. Therefore, the best method to avoid dry puffs in laboratory analysis would be to ask e-cigarette users to evaluate, by taste, what the appropriate power setting would be for a particular e-cigarette/e-liquid combination. Virtually all e-cigarette users recognize the burnt taste of a dry hit from experience [29,30]. However, human assessors may not always be available, and relying on human assessors is impractical when large numbers of devices and/or liquids need to be tested.

We therefore aimed to establish an analytical chemical method to detect dry puffs, which we calibrated with input from human assessors. A panel of highly experienced e-cigarette users first assessed the intensity of the burnt off-flavor associated with dry puffs (“dry-puff flavor”) for six different liquid/coil combinations at different power settings. Subsequently, the levels of 11 specific carbonyl compounds were measured in the emissions from the same devices, and agreed with the assessment of dry puff flavor by the human assessors.

## 2. Materials & Methods

### 2.1. Materials

Three differently flavored commercial e-liquids (vanilla, menthol and mixed fruit) and two types of coils (1.2 and 1.6 Ω) were included to test the method for different liquid/coil combinations. The vanilla and mixed fruit liquids were from the same brand and had a propylene glycol/glycerol ratio of 70/30. The menthol liquid, which was from a different brand, had a propylene glycol/glycerol ratio of 50/50. The fruit-flavored liquid contained 3 mg/mL nicotine, while the other two e-liquids did not contain nicotine (according to the labels).

The e-cigarettes consisted of JustFog Q16C clearomizers with 1.2 Ω and 1.6 Ω coils and eLeaf Pico batteries. The manufacturer indicated 3.2 V–4.4 V as a suitable voltage range for both coils, which corresponds with a power range of 6.4 W–12.1 W for the 1.6 Ω coil and 8.5 W–16.1 W for the 1.2 Ω coil. The electrical power output of the e-cigarette batteries was verified using a custom-made measuring tool and found to remain within 5% of the displayed setting (with both coils, at power levels between 5 W–25 W).

### 2.2. Recruitment and Characteristics of E-Cigarette Users

The recruitment of volunteer e-cigarette users occurred online. The study was advertised via Facebook, and via an e-mail to people that had participated in a pilot study and had expressed interest in participating in this study as well, and to people registered in a recruitment database maintained by Wageningen University (Wageningen, the Netherlands). The inclusion criteria were: (i) regular e-cigarette use (daily use of nicotine-containing e-cigarettes for at least 3 months); (ii) at least 18 years of age; (iii) no self-reported adverse health effects from vaping; and (iv) not pregnant, trying to become pregnant or lactating. The panelists confirmed (when asked during the instructions in preparation for the experiment) that they were familiar with the adverse, burnt flavor associated with dry puffs (“dry puff flavor”) from their own experience with e-cigarettes. The study was submitted to the Medical Research Ethics Committee (MREC) of Utrecht University (protocol number 18-333/C). The MREC assessment was that the study was not subject to the Dutch Medical Research Involving Human Subjects Act.

### 2.3. Sensory Assessment by E-Cigarette Users

Prior to the start of the test session, the olfactory function of each panel member was tested by a multiple-choice identification test using Sniffin’ Sticks (Burghardt, Wedel, Germany) [31]. Three of the panelists failed this Sniffin’ stick identification test (<12 correct answers out of 16) with 10, 11 and 11 correct answers, respectively. Two of them self-reported mild nasopharyngitis. However, their assessment of dry puff flavor was consistent with that of the rest of the panel and was therefore retained in the analysis (Figure S1 and Table S1). Participants were subsequently familiarized with the standardized vaping protocol and the devices used. They were given the opportunity to practice using a flavorless, nicotine-free e-liquid with a propylene glycol/glycerol ratio of 70/30.

The test sessions took place in sensory booths equipped with a computer and water tap at Wageningen University (Wageningen, The Netherlands). The room had a controlled ventilation system of five air changes per hour. Clearomizers were newly purchased and filled with e-liquid at least 3 h in advance. The airflow was set at 50%. The devices were marked with pseudo-random three-digit labels. A descriptive sensory method [32] was used for the human assessment of dry puff flavor, as follows: Experienced e-cigarette users vaped e-cigarettes at different power settings, and were instructed to report the intensity of dry puff flavor. The intensity of dry puff flavor was assessed on a 100-unit Visual Analogue Scale (VAS), with the left anchor at 0 (labelled “Not at all”) and the right anchor at 100 (labelled “So much that I would not continue vaping”). The scale was otherwise ungraduated.

Data from preliminary tests with two experienced vapers were used to establish the range of power settings that was relevant for the coils and e-liquids used in this study. These tests indicated that for all liquid/coil combinations tested, dry puff flavor was present at or below 25 W and absent at 10 W. Based on these preliminary assessments, the test started at a power setting of 10 W (which is within the range recommended by the manufacturer of the coils), and subsequently increased in 3 W steps until the dry puff flavor became so intense that the panelists reported that they would normally not have continued vaping or until a maximum of 25 W, whichever occurred first. Once the participant reported dry puff flavor, testing was stopped for that coil/liquid combination, because it was assumed that if the assessments would have continued at higher power settings, all puffs would have had dry puff flavor according to that panelist. At each power setting, the panelists were instructed to take three 3-second puffs with 1-minute intervals, cued by a visual timer on a computer monitor using Microsoft Powerpoint. A 1-minute inter-puff interval was used to allow time for the clearomizer to cool down between puffs and to prevent olfactory adaptation for the panelist [33]. Each panelist assessed the three differently flavored e-liquids using either 1.6 Ω (*n* = 7) or 1.2 Ω (*n* = 6) coils, which were assigned to the panelists at random. Panelists were allowed as much time as they wanted to recover from the previous flavor/dry puff flavor before starting with the next flavor.

Panelists were verbally instructed to disregard any personal preferences with respect to the flavors of the e-liquids (i.e., vanilla, fruit and menthol), and to report the intensity of the dry hit flavor only. Furthermore, to minimize differences in puff volume between panelists, they were instructed to vape “mouth-to-lung” (instead of “direct-to-lung”). This is a vaping technique in which users first inhale the aerosol into their mouth, and subsequently inhale it into their lungs by taking in additional air [34]. All users confirmed being familiar with this technique. This was expected to reduce the individual differences in puff volume between panelists because (1) it harmonized the vaping technique between panelists, and (2) it was expected that mouth-to-lung volumes would be less variable between participants.

### 2.4. Analysis of Carbonyl Compounds in Emissions

Emissions were collected from the devices assessed by the human panel using a Cerulean SM410 linear smoking machine (Cerulean, Milton Keynes, UK). All liquid/coil combinations were machine-vaped in the laboratory in triplicate at the same power settings assessed by the human panel (10 W–25 W in 3 W steps). A custom-made electronic timer was used to synchronize the e-cigarette battery activation buttons with the vaping machine puffs. The clearomizers were (re)filled to the maximum level at least 3 h in advance. Emissions were collected using the same topography that was imposed on the human volunteers (three 3-second puffs and 1-minute intervals). A puff volume of 55 mL was employed, approximating the volume corresponding with mouth-to-lung vaping [35].

Carbonyl compounds were analyzed as described in WHO Tobacco Laboratory Network (TobLabNet) SOP08 [36] with some modifications, as described below. Briefly, carbonyls in the emissions were trapped using a cartridge containing activated carbon (Carboxen-572, Merck Life Science NV, Amsterdam, The Netherlands) and quartz fiber filters (Cambridge Filter Pads, Borgwaldt KC, Hamburg, Germany), extracted using a mixture of carbon disulfide (CS_2_) and methanol, and subsequently derivatized with an acidified solution of dinitrophenol hydrazine (DNPH) in acetonitrile. The samples were analyzed with HPLC-DAD (high-performance liquid chromatography—diode array detection). The following modifications were made: A sample of laboratory air was collected in parallel and used to correct for the presence of small concentrations of carbonyl compounds in air.

Eleven distinct carbonyl species were identified earlier in pilot experiments (not shown). Briefly, a variety of liquids were tested at low and high power settings, and we searched for compounds that were present at high power levels but absent (or present only in very small amounts) at low power levels using various analytical techniques (e.g., GC-MS, LC-QTOF). We found that differences in the levels of carbonyls were much more pronounced than any differences we observed using other analyses. We identified 11 peaks (Table 1) that were present in e-cigarette emissions at high power levels but not at low power levels (herein referred to as “dry puff markers”). The chemical identity of most of these compounds remains unknown at present. Therefore, we referred to those compounds using their retention time. The “cutoff value” concentrations listed in Table 1 are above the limit-of-quantification, as indicated by the fact that the signal-to-noise ratio of the peaks of these eleven compounds was well above 10. Calibration samples were used to determine the concentration of formaldehyde, acetaldehyde and acrolein, as described in WHO TobLabNet SOP08 [36]. The concentration of the other compounds was estimated using the calibration curve of formaldehyde-DNPH. The absorbance of the DNPH-derivatized compounds at 365 nm was (nearly) entirely due to the DNPH chromophore. The effect of the carbonyl species on the specific absorbance was expected to be negligible. Therefore, only a small error in the absolute quantification of these compounds was expected to occur as a result of using the calibration curve of formaldehyde-DNPH for other DNPH-derivatized carbonyls, and it had no effect on the relative quantification (i.e., between samples). Data were processed using Labsolutions (Shimadzu chromatographic software, version 5.85, Shimadzu Corporation, ’s-Hertogenbosch, the Netherlands), R (v3.6.3, R Foundation for Statistical Computing, Vienna, Austria) and MS Excel (MS Excel for Office 365 v16.0, Microsoft, Redmond, WA, USA).

Several of the human panelists remarked that dry puff flavor resembles that of burnt cotton, indicating that pyrolysis of the cotton wick in the heating may contribute to dry hit flavor. Therefore, chemical analysis was also performed on the emissions from a new (unused) e-cigarette that was vaped ‘dry’, that is, in an unfilled state. For this experiment, a 1.6 Ω coil was used at a power setting of 7 W.

### 2.5. Matching Carbonyl Analysis and Dry Puff Flavor

The following steps were used to match the human assessment of dry puff flavor based on chemical analysis to the dry puff markers listed in Table 1.

(1) Each coil/liquid/power setting combination assessed by the human panel was categorized as having “dry puff flavor” or “no dry puff flavor” as follows: If more than 10% of puffs was assessed to have dry puff flavor for any coil/liquid/power setting combination, that combination was categorized as having “dry puff flavor”. In other words, with this cutoff value, no dry puff flavor was detected for 90% of puffs, even by experienced e-cigarette users. It should therefore be regarded as a pragmatic lower bound of the range of power settings employed by e-cigarette users in practice.

(2) The highest amount measured (µg/puff) of each of the dry puff markers for the coil/liquid/power setting combinations that were categorized as “no dry puff flavor” by the human panel was used as a cutoff value (µg/puff). If, for any puff, the amount of any of the 11 dry puff markers exceeded this value, it was considered a dry puff.

(3) For each of the coil/liquid/power setting combination, the emissions from three puffs from three different clearomizers were analyzed. If the amounts of any of the dry puff markers exceeded the cutoff value (i.e., exceeding the levels found in puffs categorized as not having dry puff flavor, as described in step 2) for any of the puffs from any of the three clearomizers, that power setting was considered to be too high (i.e., not user-relevant) for the coil/liquid combination.

## 3. Results

### 3.1. Assessment by E-Cigarette Users

**Figure 1:**
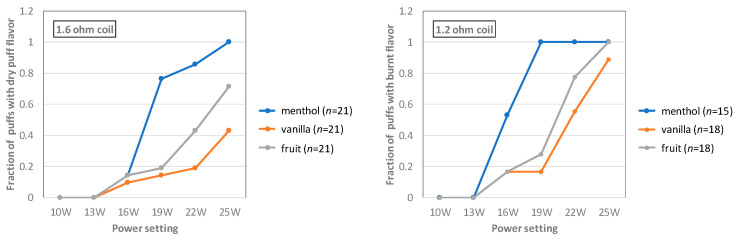
Human assessment of the presence of dry puff flavor at different power settings for each of the three e-liquid flavors tested and both coils. The vertical axis represents the fraction of all puffs for which the panelists reported dry puff flavor that was so intense they would normally not continue vaping. “*n*” represents the number of puffs assessed by the panel.

Thirteen experienced e-cigarette users (mean age 42 ± 13 year (± SD); 77% male) were included. All were former tobacco smokers (smoking for 21 ± 15 year) that had been using e-cigarettes for at least 1.5 years (5 ± 0.4 year). All participants exclusively used e-cigarettes at the time of their participation in this study (i.e., they did not also smoke tobacco cigarettes). In total, between 15 and 21 puffs were assessed by the panel for each combination of coil, flavor and power setting. This data is summarized in Figure 1.

At 10 W and 13 W, none of the panelists reported dry puff flavor for any flavor or coil. For the 1.2 Ω coil, 13 W is within the range recommended by the manufacturer (8.5 W–16.1 W). For the 1.6 Ω coil, 13 W is slightly above the range recommended by the manufacturer (6.4 W–12.1 W). Notably, at nearly every power setting above 13 W, the menthol-flavored e-liquid was reported to have dry puff flavor by a much higher fraction of the panelists than the vanilla- and fruit-flavored liquids (and the fruit-flavored e-liquid more frequently than the vanilla-flavored e-liquid).

Surprisingly, panelists using the 1.2 Ω coil reported dry puff flavor at lower power settings than those using the 1.6 Ω coil (for all three e-liquids). At the highest setting (25 W) with the 1.2 Ω coil, dry puff flavor was reported for >90% of puffs regardless of flavor, whereas with the 1.6 Ω coil, this was only the case for the menthol-flavored e-liquid. For the fruit- and vanilla-flavored e-liquids, even at the highest power setting, only 71% and 42% of puffs were reported to have dry puff flavor with the 1.6 Ω coil.

### 3.2. Analysis of Carbonyl Compounds in Emissions

**Figure 2:**
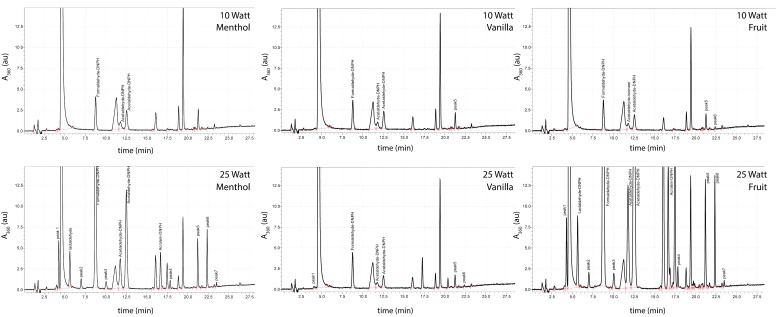
HPLC-DAD (λ = 360 nm) chromatograms of DNPH-derived carbonyl compounds in e-cigarette emissions for the three different e-liquids at 10 W and 25 W power using a 1.6 Ω coil. The two peaks named “acetaldehyde-DNPH” are isomers. Relevant peaks of unknown chemical identity are labeled “peak1” to “peak7”.

Figure 2 shows typical chromatograms for the three different e-liquids examined at the lowest (10 W) and highest (25 W) power settings. The concentration of the 11 dry puff markers was found to increase strongly at high power settings.

### 3.3. Matching Dry Puff Flavor to Carbonyl Analysis

**Figure 3:**
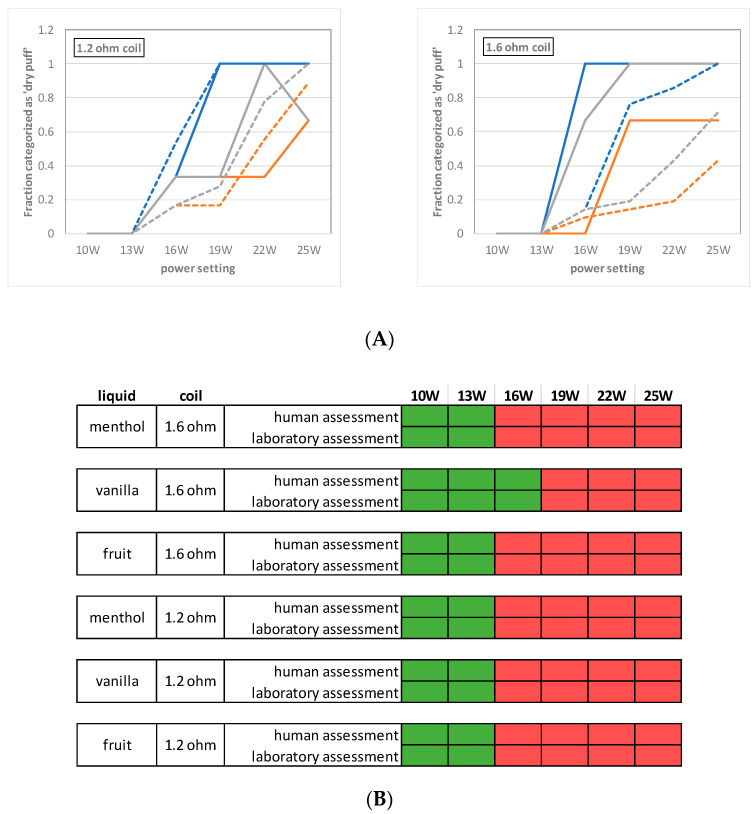
(**A**): Dry puff flavors of human assessment (dotted lines; these are the same data as shown in Figure 1) compared with the categorization based on laboratory analysis of dry puff markers (solid lines). Colors indicate flavor: menthol:blue, vanilla:orange, fruit:grey. At 25 W using the 1.2-ohm coil and the fruit-flavored e-liquid, one of the three laboratory measurements yielded unexpectedly low amounts of the dry hit markers, resulting in the unexpected “drop-off” of the graph from 22 W to 25 W. This was likely caused by a technical problem with the e-cigarette or vaping machine that occurred during this measurement, but the data were nevertheless retained because the reason is unknown. (**B**): Suitability of different power settings for each coil/liquid combination, as assessed by human e-cigarette users and based on the amounts of dry puff markers measured in the laboratory. For the “human assessment”, red indicates that 10% or more of the puffs for that liquid/coil/power setting combination were reported by the e-cigarette user panel to have dry puff flavor. For the “laboratory assessment”, red indicates that one or several dry puff markers exceeded its cutoff value (Table 1) for one or more of the three puffs analyzed.

Using the cutoff values in Table 1, we categorized the puffs analyzed in the laboratory into “dry puff” or “no dry puff” categories. For each coil/liquid/power setting combination, the fraction of puffs that was categorized as dry puffs is shown in Figure 3A.

Using this simple algorithm (described under “Matching carbonyl analysis and dry puff flavor”), the categorization based on the chemical analysis of dry puff markers matched the human assessment for 83% of all puffs (*n* = 108, false-negative rate (FN): 17%, false-positive (FP): 0%). Of the individual dry puff markers, lactaldehyde matched best with the human assessment of dry puff flavor (78% of all puffs match, FN 22%, FP 0%).

Subsequently, we categorized the different coil/liquid/power setting conditions based on the three puffs analyzed in the laboratory (Figure 3B).

The menthol-flavored e-liquid was expected to exhibit dry puff flavor more frequently and at lower power settings than the fruit and vanilla-flavored e-liquids, in line with the human assessment. Similarly, at nearly all power settings above 13 W, the fruit-flavored e-liquid was classified more frequently as “expected to have dry puff flavor” than the vanilla-flavored e-liquid.

At 10 W and 13 W, none of the measurements were classified as “expected to have dry puff flavor”, which also agrees with the observations by the human panel. For the vanilla-flavored e-liquid using the 1.6-ohm coil, a power setting of 16 W was assessed by the human panel as having no dry puff flavor, which agreed with the expectation based on the carbonyl analysis.

## 4. Discussion

In this study, we established an agreement between the levels of 11 carbonyl compounds in the emissions of e-cigarettes and the presence of dry puff flavor according to a human panel consisting of experienced e-cigarette users. Therefore, this set of carbonyl compounds is very useful for detecting and avoiding dry puffs when collecting e-cigarette emissions with a laboratory vaping machine. This approach is easier, faster and more ethical than using human test subjects to assess dry puff flavor. Although it would be preferable to measure these 11 compounds for each liquid/coil combination being tested (rejecting any measurements in which one of the carbonyl levels exceeds its cutoff value), this would be a time-consuming approach. Instead, a more pragmatic approach might be adopted by testing a sample of several different e-liquids with a particular device. The highest power setting at which all e-liquids can be vaped without producing dry hits can then be used to test other e-liquids.

These 11 “dry puff markers” can be conveniently quantified using TobLabNet method SOP08 [36], which is a simple and well-described method for the analysis of carbonyl compounds in tobacco products. The human assessment of dry puff flavor from the chemical data was related to the laboratory results using a simple algorithm based on a cutoff value for each compound.

Using only 1 or a few of the dry hit markers to detect dry puffs, rather than all 11, might seem more convenient. However, the use of all 11 markers together improves the robustness of the assay with respect to analytical variation between the measurements and differences between e-liquids, and the sensitivity to dry hits. Considering that all 11 compounds were measured in 1 run in the same assay, the analysis of all markers can be efficient. Of the individual dry puff markers, lactaldehyde was found to provide the best indication of dry puffs.

### 4.1. Panel Assessment Cutoff Value Substantiation

The 10% cutoff value that was used to discriminate between the assessments of the human panel as “dry puff flavor”/“no dry puff flavor” should be considered a pragmatic lower bound of the range of user-relevant power settings. At this cutoff value, the human panel reported the dry puff flavor for only 10% of the puffs they assessed. In other words, the flavor of the vast majority of puffs (90%) was considered acceptable to experienced users, and they would have normally continued vaping at this power setting. Therefore, the cutoff values in Table 1 are conservative in that they ensure that even subtle dry puff flavor (unnoticeable to 90% of experienced users) is avoided. For assessing the composition of e-cigarette emissions under conditions that are relevant to a majority of users (>50%), different cutoff values would be more appropriate.

### 4.2. Flavor Preference

The volunteers in our study were instructed to disregard their own personal preferences with respect to the intended flavor of the e-liquids (i.e., vanilla, menthol and fruit), and to assess only the dry puff off-flavor. It is unclear to what extent the (untrained) panelists were able to do this. One panelist did not like the menthol flavor and did not asses it. We cannot exclude that the panelists may have found the dry puff flavor more acceptable if they liked the intended flavor of a particular e-liquid, or that dry puff flavor might have been masked by the e-liquid flavor. Although this also occurs in real life, and is therefore, in principle, accounted for in the assessment, it does raise the question of whether our group of participants was large enough to represent the variation in flavor preferences among e-cigarette users.

### 4.3. Causal Relation between Dry Puff Flavor and Chemical Markers

It is unknown exactly what compound(s) cause the adverse flavor that human e-cigarette users experience during dry puff conditions. Most likely, it is a compound or a combination of compounds that form by the thermal decomposition of the e-liquid or wick. Many of such compounds have been identified [17,21], but it remains unknown to what extent they contribute to dry puff flavor. Several of the panelists remarked that dry puff flavor resembles that of burnt cotton, which suggest that decomposition of the cotton wick may contribute to generation of the dry puff flavor. Two observations in this study support this. First, several of the compounds associated with the dry puff markers were observed independently of which e-liquid was tested, suggesting that they originate from decomposition of the wick or a common component of the three liquids tested (such as propylene glycol or glycerol). Second, dry puff markers were also observed in the emissions from an unfilled e-cigarette, i.e., an e-cigarette that was vaped dry (without e-liquid). The unfilled e-cigarette was indeed classified as “expected to have dry puff flavor” based on the carbonyl content in its emissions (all 11 dry puff markers exceed their cutoff values). This suggests that (some) of the dry puff markers identified in this study might form by decomposition of the cotton wick. Because of the possible contribution of the wick to dry puff flavor, it is important to investigate in future studies whether dry puff flavor can be detected by the described method in atomizers that utilize other wick materials, such as silica fibers or ceramics.

### 4.4. Agreement for Different Liquids and Coils

Interestingly, the assessment by the human panel and the expectation of dry puff flavor based on the analytical chemical method agree that there are differences between the e-liquids tested. The menthol-flavored e-liquid frequently yielded dry puff flavor at intermediate power settings (16–19 W) in contrast with the vanilla- and fruit-flavored liquids. It should be noted that the menthol-flavored liquid was from a different brand and had a different propylene glycol/glycerol ratio (50/50) than the other two liquids (70/30). Possibly, differences in viscosity or the presence of thermally unstable ingredients contributed to its apparent liability to dry puffs. Sensory effects other than olfactory perception may also have played a role in the human assessment of the different e-liquids, such as the cooling effect of menthol and the higher temperature of the aerosol at high power settings. It is also conceivable that the (intended) flavor of the e-liquid masks the dry puff flavor or allows the dry puff flavor to be recognized more easily.

### 4.5. Future Research

The chemical identity of the majority of the dry puff markers was not resolved. This does not preclude the relative quantification of the dry puff markers between samples, but it would be of interest to identify these compounds. First, it would allow the use of chemically pure standards for absolute quantification, improving the repeatability between laboratories and instruments. In addition, it would allow the development of optimized methods for measuring these compounds. This might also shed more light on the chemical processes that give rise to the dry puff flavor.

To test the validity and repeatability of the described method, the sensory analysis may be repeated with another cohort of e-cigarette users to confirm that the results are not only valid for the current panel.

Furthermore, it would be interesting to examine to what extent the described method applies to other e-liquids, with other flavors and ingredients, and to devices with different specifications and designs (e.g., ceramic coils, silica wicks, sub-Ω coils).

Finally, it would be interesting to study the effects of age and gender on the ability of e-cigarette users to notice the dry puff flavor. Olfactory performance tends to decrease with age, and there is evidence to suggest that sex differences exist in olfactory performance. However, a much larger sensory panel would be required for this purpose.

### 4.6. Practical Implications

It is essential to use human-relevant conditions when generating e-cigarette emissions for analysis, for instance, when assessing the health risks of e-cigarettes based on the levels of their toxic emissions, or to allow regulation of product standards. Many studies have shown that the power setting of an e-cigarette is a critical parameter in this respect [17,18,19,20,21,22,23]. Farsalinos et al. [26] reported that formaldehyde, acetaldehyde and acrolein levels were raised by 30–250 times when using excessively high power settings. To date, however, no methods have been described to select an appropriate power setting except by human assessment of the flavor. The method described in this study offers a tool for researchers to check if an e-cigarette power setting for a particular liquid/coil combination is not excessively high (i.e., not representative of e-cigarette user behavior) using only analytical chemical analysis. This should improve the relevance of measurements of e-cigarette emissions, enabling more accurate risk assessments while avoiding the need for human volunteers to asses e-cigarette power settings.

## 5. Conclusions

Dry puffs can be detected by the chemical quantification of 11 carbonyl compounds in the emission of e-cigarettes. We recommend that measurements of these dry puff markers are routinely performed when analyzing e-cigarette emissions for risk assessment and regulation to avoid measuring unrealistically high levels of toxicants.

## Figures and Tables

**Table 1:** Retention times (Rt) of the 11 “dry puff markers” (using WHO TobLabNet SOP08 [36].) and the highest amounts measured for liquid/coil/power settings combinations categorized as “no dry puff flavor” based on the results of the human assessors (“cutoff values”). Peaks corresponding to these compounds were identified in the chromatographic data by matching the retention time listed here to the retention time of peaks in the chromatogram. The concentration of each compounds was taken to be proportional to its peak surface area. Quantities in µg were derived from the surface areas using formaldehyde-DNPH as a standard.

Compound	Rt ^1^ (min)	Cutoff Values (µg/puff)
peak1	4.29	1.2
lactaldehyde-DNPH ^2^	5.64	0.8
peak2	7.01	0.3
formaldehyde-DNPH	8.78	6.5
peak3	10.05	0.3
Acetaldehyde-DNPH (isomer 1)	11.78	6.7
Acetaldehyde-DNPH (isomer 2)	12.52	
acrolein-DNPH	16.68	0.4
peak4	17.85	0.2
peak5	21.21	1.9
peak6	22.36	1.4
peak7	23.50	0.2

^1^ Rt—retention time; ^2^ DNPH—dinitrophenyl hydrazine.

## Supplementary Materials

The following are available online at https://www.mdpi.com/article/10.3390/ijerph182111520/s1, Figure S1: Effect of excluding participants that failed the Sniffin’ stick identification test. Table S1: Demographic data of panel participants.
