# Supplementary material for: Improving the Analysis of E-Cigarette Emissions: Detecting Human “Dry Puff” Conditions in a Laboratory as Validated by a Panel of Experienced Vapers
Source: Int J Environ Res Public Health. 2021 Nov 2;18(21):11520. doi: 10.3390/ijerph182111520 (PMC8583459; doi:10.3390/ijerph182111520)
Supplement: Supplementary file 1 [file ijerph-18-11520-s001.zip › ijerph-1353262-supplementary.pdf]

Supplementary Info S1

As explained in the main text, three of the 13 panelists failed the Sniffin' stick identification test (<12 correct answers out of 16) with respectively 10, 11 and 11 correct answers. Two of them self-reported mild nasopharyngitis. However, their assessment of dry puff flavor was consistent with that of the rest of the panel and was therefore retained in the analysis.

This Supplementary Figure S1 shows what the effect would be if the data from these 3 panelists is removed from the analysis. Comparing this Supplementary Figure S1 with Figure 1 in the main text, it is clear that there are no important differences.

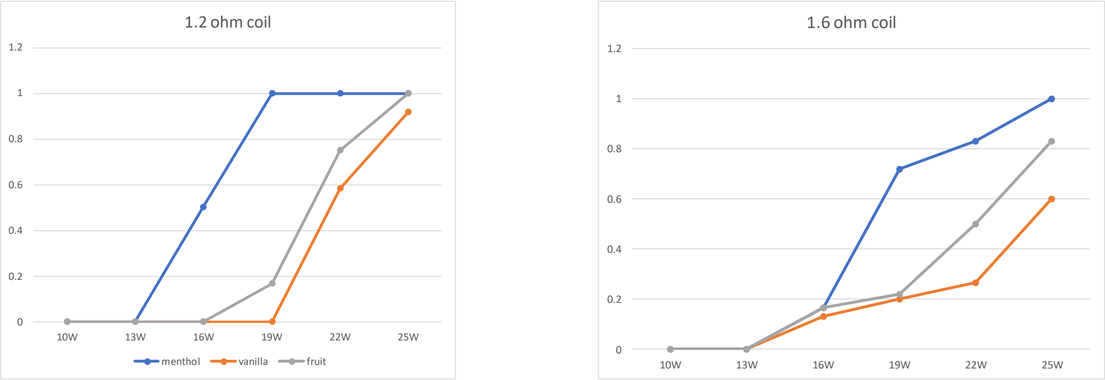

Supplementary Figure S1: Effect of excluding participants that failed the Sniffin' stick identification test. Shown in this figure is the human assessment of the presence of dry puff flavor at different power settings, for each of the three e-liquid flavors tested, as in figure 1 in the main text. However, in contrast to figure 1 in the main text, only data from panelists that passed the sniffin' stick identification test were used for this figure. The vertical axis represents the fraction of all puffs for which the panelists reported dry puff flavor that was so intense they would not normally continue vaping.

Supplementary Table S1

Supplementary Table S1: Demographic data of panel participants

participant	age (years)	sex (male/female)	number of years of smoking	number of months of e-cigarette use	brand/model of own e-cigarette	prefered flavor
1	38	M	14	36	Dotmod box 200W RTA	custard
2	66	F	50	36	zensations en just-fog	tobacco
3	51	F	25	120	eleaf melo 4 tank / eleaf ikuu 200 MOD	menthol
4	18	F	1	18	smok	kiwi/strawberry and juicy peach
5	49	M	20	24	bookwill	cola, fruit, apple, watermelon
6	26	M	4	60	zenith 0.5 ohm	forest-fruits
7	27	M	2	18	joyetech exceed & Innokon Z-biip	mint
8	46	M	25	84	Squid double barrel + drop dead	Bakery / Custard
9	46	M	30	84	Justfog C14 standaard	tobacco
10	34	M	11	84	aramax power	energy, melon
11	48	M	35	72	box mod	tobacco
12	50	M	35	36	Smok Species max 130 W	Menthol and Cappuccino
13	48	M	27	102	FFV16-SMOK	fruit flavors
